# Development of a low-cost culture medium for the rapid production of plant growth-promoting *Rhodopseudomonas palustris* strain PS3

**DOI:** 10.1371/journal.pone.0236739

**Published:** 2020-07-30

**Authors:** Kai-Jiun Lo, Sook-Kuan Lee, Chi-Te Liu

**Affiliations:** 1 Institute of Biotechnology, National Taiwan University, Taipei, Taiwan; 2 Agricultural Biotechnology Research Center, Academia Sinica, Nankang, Taipei, Taiwan; Luleå University of Technology, SWEDEN

## Abstract

*Rhodopseudomonas palustris* PS3 is one of the purple phototrophic non-sulfur bacteria (PNSB), which have plant growth-promoting effects on various plants. To expand the scale of PS3 fermentation in a time- and cost-effective fashion, the purpose of this work was to evaluate the use of low-cost materials as culture media and to optimize the culture conditions via response surface methodology. Corn steep liquor (CSL) and molasses were identified as potential materials to replace the nitrogen and carbon sources, respectively, in the conventional growth medium. The optimum culture conditions identified through central composite design were CSL, 39.41 mL/L; molasses, 32.35 g/L; temperature, 37.9°C; pH, 7.0; and DO 30%. Under the optimized conditions, the biomass yield reached 2.18 ± 0.01 g/L at 24 hours, which was 7.8-fold higher than that under the original medium (0.28 ± 0.01 g/L). The correlation between the predicted and experimental values of the model was over 98%, which verified the validity of the response models. Furthermore, we verified the effectiveness of the *R*. *palustris* PS3 inoculant grown under the newly developed culture conditions for plant growth promotion. This study provides a potential strategy for improving the fermentation of *R*. *palustris* PS3 in low-cost media for large-scale industrial production.

## Introduction

*Rhodopseudomonas palustris* is one of the purple phototrophic non-sulfur bacteria (PSNB). Since it can process nutrients through various metabolic pathways, such as photosynthetic, photoheterotrophic, chemoheterotrophic and chemoautotrophic pathways, *R*. *palustris* can inhabit a variety of environments [1]. It is already known that *R*. *palustris* contains many useful characteristics and is broadly used in industry for bioremediation, sewage treatment, removal of phytotoxic compounds, etc. [2, 3]. This strain is able to convert complex organic compounds into biomass and bioenergy, regardless of whether the substrates are plant-derived, pollutant or aromatic compounds [1, 4–7]. *R*. *palustris* has also been reported to act as a promising biofertilizer for promoting crop yield and improving soil fertility [8–10]. Previously, we isolated an *R*. *palustris* strain, called as the PS3, from Taiwanese paddy soil that *not only had beneficial* effects on plant growth but also enhanced the efficiency of the uptake of the applied fertilizer nutrients [8, 11]. Accordingly, this strain has been considered an elite microbial inoculant for agricultural applications.

For the purpose of the commercialization or the large-scale field application of the selected microbes, fermentation production is an essential prerequisite. The yield per unit biomass of the mass production is influenced by several factors, such as the medium composition (carbohydrate and nitrogen sources, minerals, etc.), the culture conditions (pH, temperature, agitation and aeration, etc.) and the mode of fermentation (batch, fed-batch and continuous fermentations) [12]. The scaling-up of microbial processes is commonly undertaken in lab-scale process development with expensive medium components such as yeast extract, beef extract, and peptone [13]. These high-cost media result in a limitation on commercialization. To reduce the cost of fermentation, complex raw materials derived from plant and animal residues, as well as from agricultural and food industrial wastes, are mainly used [14]. For example, cheese whey, corn steep liquor (CSL), corn syrup, distillery yeast, molasses, soybean and starch are widely applied [15–19]. These relatively inexpensive raw materials have already been used as suitable nutrients for ensuring the growth of bacteria and for the production of different primary and secondary metabolites [17, 20–22].

Response surface methodology (RSM), also called the Box–Wilson methodology, is a useful tool to optimize the factors of fermentation processes by applying mathematics and statistics [23]. This technique is an experimental design based on the fit of a polynomial regression model for testing the multiple factors that influence the responses by varying the factors simultaneously, and fewer experiments are needed to study the effects of all factors [24, 25]. Through RSM, the interaction effects of individual factors can also be determined. The common experimental designs for RSM are central composite design (CCD) and Box-Behnken design (BBD). Although BBD has been suggested to reduce the number of trials required for a large number of variables, this advantage will disappear when in four or more factors [23]. Moreover, because CCD consists of a factorial or fractional factorial design with center points augmented with a group of axial points, it is often applied in sequential experiments and can be used in the estimation of model curvature [23]. Overall, the RSM procedure includes the selection of independent variables, the delimitation of the experimental factor region, the evaluation of the model’s fitness, and finally, the attainment of optimum values and verification [23, 24].

The van Niel medium and modified van Niel medium have been designed for *R*. *palustris* fermentation [26, 27]. Du et al [28] cultivated *R*. *palustris* strain DH with the original van Niel medium for 7 days, and the derived biomass was ~0.3 g/L. Carlozzi and Sacchi [29] incubated *R*. *palustris* strain 42OL in a modified van Niel medium to which sodium acetate was added, and the derived biomass was 1.42 g/L/d. Chang et al [30] modified the original medium with sodium acetate and peptone, and the derived biomass of *R*. *palustris* strain YSC3 was 0.36 g/L after 2 days of incubation. However, the use of these media may be uneconomical for industrial application due to their high cost and long incubation time. The cost is primarily due to expensive nitrogen sources, such as yeast extract and peptone. On the other hand, some media have low-cost sources; for example, Xu et al [31] used *Stevia* residue extractions supplied with NH_4_Cl as a medium to culture *R*. *palustris*, and the highest obtained biomass was 1.5 (OD_660_) (approximately equivalent to 0.77 g/L) after 96 hours of cultivation. Kornochalert et al [9] obtained the 0.9 g/L *R*. *palustris* in latex rubber sheet wastewater supplied with pineapple extract after 96 hours of fermentation. In our previous study, we used a modified van Niel medium with malate and yeast extract as the carbon and nitrogen sources for the fermentation of *R*. *palustris* PS3, and the biomass was 0.07 g/L after 24 hours of cultivation. Although this broth showed promising plant growth-promoting effects on several crops [8, 11, 32], the cost was too high for industrial application. Therefore, the purpose of this study was to develop an optimal fermentation protocol for *R*. *palustris* PS3 that is cost-effective, results in high biomass yield, and has a short fermentation time. We evaluated some agro-industrial by-products, including corn steep liquor (CSL), beetroot extract (BRE), soybean flour (SF), soybean protein isolate (SPI), molasses and corn starch, for use as substrates for *R*. *palustris* PS3 cultivation in terms of cost reduction and tested them with RSM to obtain the optimal fermentation conditions. Furthermore, we inoculated the newly developed fermentation broth into plants to verify its plant growth-promoting effect.

## Materials and methods

### Microorganism and bacterial preparation

*R*. *palustris* PS3 was isolated from Taiwanese paddy soil and showed promising beneficial effects on plant growth in our previous studies [8, 11]. This bacterium was grown in 3 mL of modified van Niel medium [8] (designated PNSB medium hereafter) at 37°C and 200 rpm. The PNSB medium consisted of KH_2_PO_4_ 1.0 g/L, NH_4_Cl 1.0 g/L, MgSO_4_•7H_2_O 0.2 g/L, FeSO_4_•7H_2_O 0.01 g/L, CaCl_2_ 0.02 g/L, MnCl_2_•4H_2_O 0.002 g/L, Na_2_MoO_4_•2H_2_O 0.001 g/L, yeast extract 0.5 g/L, and malate 5.0 g/L, pH = 7.0. After 24 hours incubation, 2 mL of the above bacterial broth was incubated in a 250 mL Erlenmeyer flask containing 50 mL PNSB medium and then cultured at 37°C with shaking at 200 rpm. After 24 hours incubation, the bacterial broth was diluted with fresh PNSB medium and adjusted to an optical density approximately equal to OD_600_ = 1.0. This diluted bacterial broth was used as a seed culture for further experiments.

### Screening of nitrogen and carbon sources for medium optimization

Different nitrogen (yeast extract, NH_4_Cl, corn steep liquor (CSL) (TAIROUN PRODUCTS Co., Ltd., Taiwan), beetroot extract (BRE) (Hauert HBG Dünger AG, Switzerland), soybean flour (SF), soybean protein isolate (SPI) and NH_4_NO_3_) and carbon (malate, sodium acetate, glucose, fructose, molasses (TAIWAN SUGAR Co. Ltd., Taiwan) and corn starch (TAIROUN PRODUCTS Co., Ltd., Taiwan)) sources were used to optimize the composition of the PNSB medium. The initial concentrations of the individual carbon and nitrogen sources followed those in the PNSB medium described above and were sterilized separately by autoclave. For preliminary screening of alternative nitrogen sources, a final concentration of 1.5 g/L of each nitrogen-containing candidate material was introduced to substitute NH_4_Cl (1.0 g/L) as well as yeast extract (0.5 g/L) in the presence of 5.0 g/L malate (carbon source). For preliminary screening of alternative carbon sources, a final concentration of 5.0 g/L of each carbon-containing candidate material was introduced to substitute malate (5.0 g/L) in the presence of CSL (1.5 g/L) as the selected nitrogen source.

For the experiments, the seed culture mentioned above was centrifuged at 3,000 rpm for 5 min at 4°C and then suspended in phosphate buffer solution (PBS). The final OD_600_ was adjusted to 1.0, inoculated (10% v/v) into 50 mL of the respective modified media in a 250 mL Erlenmeyer flask, and then cultured at 37°C with shaking at 200 rpm.

### Measurement of cell growth

The population of PS3 cells was estimated by a standard plate count method. Enrichment culture broth was serially diluted by fresh PNSB medium, and spread onto PNSB agar plate. Incubated the plate at 37°C in the dark and then calculated the number of colony forming unit (CFU) per milliliter. On the other hand, the cell concentration was also measured by optical density with a spectrophotometer at 600 nm. The bacterial culture broth was diluted in distilled water to obtain an optical density less than 0.6, and the OD_600_ was multiplied by the dilution times. Biomass (grams per liter) was assayed from the OD_600_ value by using a calibration standard curve. Viable counts were determined by the plate counting method.

### Construction of the response surface model by central composite design (CCD)

Based on the results of factor screening, the following independent variables, including the medium components (molasses and CSL) as well as the fermentation conditions (temperature and pH), were selected for optimization by central composite design (CCD). The CCD consists of three parts: a factorial design, a central points, and axial points [23]. In order to reduce the experimental trials, fractional factorial design was substituted for factorial design. For CCD construction, we performed a 2^4−1^ (four factors) fractional factorial design (FFD) using R software with the package “rsm”. Then, the design was augmented by eight axial points and four replications of the center points. This design resulted in a total of 20 experiments [33]. The distance of the axial points (1.682) from the central point was developed by the software with the default settings (rotatable if possible) [33]. Before performing a regression analysis, the factors will be normalized. Codification of the levels of variables needs transforming the real studied value to the range without dimension (-1 to +1). The various factors were coded according to the following equation:

$$x_{i}\text{=}\frac{E_{i}-E_{0}}{\Delta E_{i}}$$ Eq (1)

where *x*_*i*_ is the coded variable of the factor, *E*_*i*_ is study value of variable, *E*_0_ is the real value of the variable at the center point, and Δ*E*_*i*_ is the step-altered value, which represents the difference from the real value in the higher or lower value from the real value at the central point. In present study, the Δ*E*_*i*_ for CSL, molasses, temperature and pH value are 5 mL/L, 4 g/L, 1.5°C and 0.3, respectively. The various factors and levels for the CCD are shown in Table 1. The matrix corresponding to the CCD and the total experimental data from 20 runs are shown in S1 Table in S1 File. The trail no. 1 to 8 represented fractional factorial design (FFD); trail no. 9 to 12 were attributed to center point; and trail no. 13 to 20 were referred as axial points. The experimental data of the CCD were fitted with a quadratic second-order polynomial equation by a multiple regression technique. The regression equation is shown as Eq (2):

$$Y=\beta_{0}+\sum_{i=1}^{k}\beta_{i}x_{i}+\sum_{i=1}^{k}\beta_{ii}x_{i}^{2}+\sum_{i=1}^{k-1}\sum_{j=1+i}^{k}\beta_{ij}x_{i}x_{j}$$ Eq (2)

where *Y* is the predicted response, *β*_0_ is the intercept, and *β*_i_, *β*_ii_ and *β*_ij_ are the linear, quadratic and cross-interaction regression constant coefficients, respectively. *x*_*i*_ and *x*_*j*_ are the coded independent variables for factors. The quality of the fitting of the second-order equation model to the data was described by the coefficient of determination R-squared, and the statistical significance was evaluated by the F-test. The significance of the regression coefficients was analyzed by a t-test. The computer software used was R, version 3.6.2 [34].

**Table 1:** Level and code of variables in the CCD experiments.

Independent variable	Coded levels				
	-1.682	-1	0	1	1.682
Corn steep liquor (mL/L)	31.59	35	40	45	48.41
Molasses (g/L)	28.27	31	35	39	41.73
Temperature (°C)	35.9	37	38.5	40	41
pH	6.06	6.4	6.9	7.4	7.74

The various factors were coded according to Eq (1).

### Batch-culture experiments in a benchtop bioreactor

To perform the response surface methodology assay, a 5-L stirred tank bioreactor (BTF-A5L, BIOTOP Inc, Taiwan) was used to carry out all of the batch-cultur**e** experimental trials. We inoculated 300 mL of bacterial culture into 3 L of fresh medium. The conditions for molasses, CSL, temperature and pH value were set according to the experimental design matrix corresponding to the CCD described. The pH value of the cultures was controlled by pH-stat and automatically adjusted with 2 N NaOH and 2 N HCl.

### In planta experiments to verify the plant-growth promotion effect of the newly developed fermentation broth

Chinese cabbage seeds (*Brassica rapa* L. spp. *pekinensis* cv. “Michelle”) were purchased from Formosa Farming Materials Co., Ltd. (Taipei, Taiwan). The seeds were immersed in 70% alcohol for 3 min and then in 3% hydrogen peroxide solution for 7 min for surface sterilization, followed by a thorough washing with sterile distilled water. These seeds were germinated for 1 day at 25°C in the dark. For hydroponic cultivation, well-germinated seeds were transferred to completely wet cotton and cultivated under continuous (24-h photoperiod) light-emitting diode (LED) light (~210 μmol m^-2^s^-1^). After one week, the seedlings were transferred to hydroponic tanks (35 L) in a plant factory facility (College of BioResources and Agriculture, National Taiwan University). Twenty-four seedlings were cultivated in each tank, and each tank was equipped with an air pump to homogenize the solution and maintain the dissolved oxygen. Hoagland’s solution (0.51 g L^−1^ KNO_3_, 0.49 g L^−1^ MgSO_4_•7H_2_O, 0.08 g L^−1^ NH_4_NO_3_, 0.068 g L^−1^ KH_2_PO_4,_ 22.5 mg L^−1^ Fe-EDTA, 2.86 mg L^−1^H_3_BO_,_ 0.051 mg L^−1^ CuSO_4_, 0.22 mg L^−1^ ZnSO_4_•7H_2_O, 1.81 g L^−1^ MnCl_2_•4H_2_O, 0.12 mg L^−1^ Na_2_MoO_4_•2H_2_O and 1.18 g L^−1^ Ca(NO_3_)_2_•4H_2_O) was used as a nutrient sources [35]. The concentrations of the hydroponic nutrient solution were measured by an electrical conductivity meter (EC meter) (Spectrum® Technologies, Inc.) and adjusted with concentrated hydroponic nutrient solution to maintain an EC value of 1.2–1.3 dS m^-1^. The initial pH value of the hydroponic nutrient solution was adjusted by H_3_PO_4_ and 2N KOH to 7.0. The cultivation environment was set at 25°C, 70% humidity, and 210 μmol.m^-2^.s^-1^ light intensity for 16 hours. We poured 250 mL of the liquid culture (OD_600_ = 0.1, equivalent to ~ 10^8^ colony-forming units [CFU]/mL) into the tank, resulting in a final bacterial concentration of 10^6^ CFU/mL. After seven days post inoculation (7 dpi), another 250 mL of the liquid culture was applied to each tank. Seventeen days after planting (DAP), the Chinese cabbages were harvested, and the fresh and dry weights were measured. For soil cultivation, well-germinated seeds were transferred to cultivatable soil under controlled illumination with a 16-hour photoperiod (~210 μmol m^-2^s^-1^). After one week, the seedlings were transferred to pots containing approximately 300 g cultivatable soil. The cultivation environment was set to be consistent with that under hydroponic cultivation. Two milliliters of the respective culture broth (OD_600_ = 0.1, equivalent to ~ 10^8^ colony-forming units [CFU]/mL) and 0.05g chemical fertilizer were added into each pot once a week as described previously [8]. The chemical fertilizer was purchased from SINON Co., Ltd. (New Taipei City, Taiwan) which consists of 14% ammonium nitrogen, 15% citric acid-soluble Phosphorus (13.5% water-soluble phosphorus), and 10% water- soluble potassium. At 28 DAP, the Chinese cabbages were harvested, and their fresh and dry weights were measured.

### Statistical analysis

Analyses of variance (ANOVA) were performed with R version 3.6.2 [34]. Fisher’s least significant difference (LSD) test was used for multiple range analyses to determine the significant differences between the groups of data. The results were considered significant at P = 0.05.

## Results

### Selection of appropriate components for optimization of *R*. *palustris* strain PS3 medium

**Fig 1:**
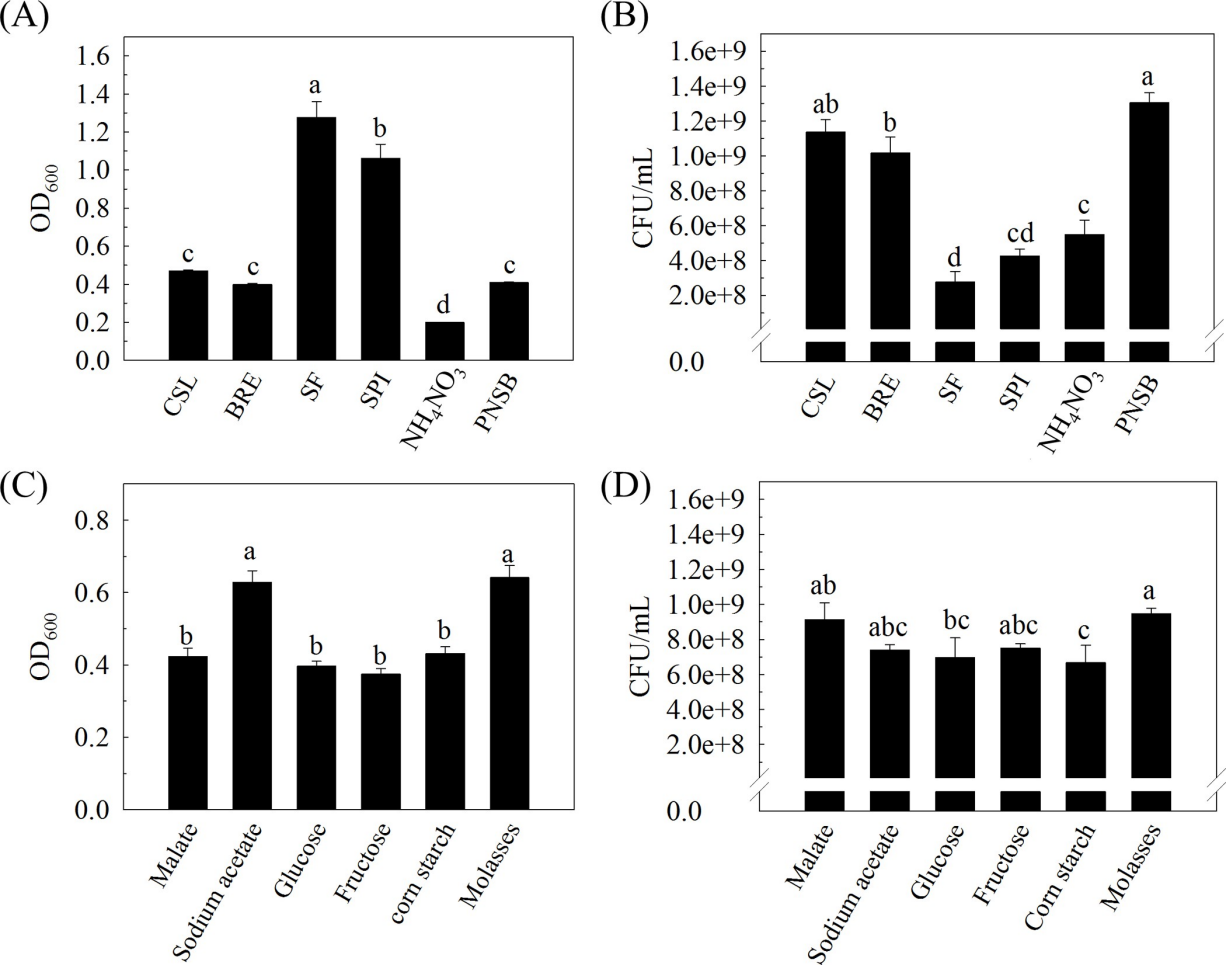
Selection of appropriate components for an optimized *R*. *palustris* medium. The effect of different nitrogen (A, B) and carbon sources (C, D) on the growth of *R*. *palustris* PS3 culture under 200 rpm and 37°C for 24 hours. In the nitrogen source screening experiments (A and B), each nitrogen source (1.5 g/L) was used to replace 1.0 g/L NH_4_Cl and 0.5 g/L yeast extract in the PNSB treatment. In the carbon source screening experiments (C and D), 1.5 g/L CSL was used as the nitrogen source in the medium, and the concentration of each carbon source was set at 5 g/L. CSL: corn steep liquor; BRE: beetroot extract; SF: soybean flour; SPI: soybean protein isolate; PNSB: modified van Niel medium [8]. Vertical bars represent the standard error of each mean, and bars with different letters indicate statistically significant differences at P = 0.05 according to LSD test.

Carbon and nitrogen sources provide important nutrients for bacterial growth. To screen suitable carbon and nitrogen sources for viable PS3 cell production, a “one-factor-at-a-time” method (OFAT, or single-variable *optimization* strategy) was used. We selected corn steep liquor (CSL), beetroot extract, soybean flour (SF), soybean protein isolate (SPI), and NH_4_NO_3_ as the individual nitrogen sources and malate, sodium acetate, glucose, fructose, molasses, and corn starch as the individual carbon sources. These materials are common, low-cost agro-industrial byproducts and are the most common nutrient sources in industrial fermentations [14, 36]. Fig 1 shows the effect of the different selected nitrogen and carbon sources on the growth of the *R*. *palustris* PS3 strain. In the presence of 5 g/L malate as single carbon source, the highest turbidity (OD_600_) was observed when using SF or SPI as the sole nitrogen source, followed by that of the strain incubated with CSL, BRE and PNSB and that from fermentation in NH_4_NO_3_ (Fig 1A). To estimate the cell concentration of the PS3 culture, we also verified the correlation between turbidity (OD_600_) and colony-forming unit (CFUs) counts. As shown in S1 Fig in S1 File, the OD_600_ and CFUs values were positively correlated during the fermentation progress (24 hr). As shown in Fig 1B, when using CSL as the sole nitrogen source, the cell viability of *R*. *palustris* PS3 was 1.14 ± 0.72 × 10^9^ CFU/mL, which was comparable to that with the original nitrogen source (NH_4_Cl and yeast extract in PNSB medium) and higher than with the other nitrogen sources. Although the soybean flour (SF) and soybean protein isolate (SPI) also showed high OD_600_ values, their CFUs were dramatically lower than the others. This implied that the turbidities (OD_600_) of these two microbial samples did not reflect the number of viable cells produced (CFU). This inconsistency may be attributed to their poor solubility in the individual media, which interfered with the turbidity readout. Accordingly, CSL is considered a potential nitrogen source for the production of *R*. *palustris* PS3. After 1.5 mL/L CSL was determined as the nitrogen source for the modified medium, we further screened appropriate carbon sources. We found that molasses and sodium acetate significantly increased the biomass of *R*. *palustris* PS3 compared with those under the other carbon sources (Fig 1C), with OD_600_ values of 0.641 ± 0.033 and 0.628 ± 0.032, respectively. On the other hand, higher viable cell counts were present in the molasses and malate treatments than in the other treatments, and the biomass was approximately 10^9^ CFU/mL (Fig 1D). We noticed that when sugars were used as carbons (i.e., glucose and fructose), the corresponding cell viability (CFU/mL) was significantly lower than that resulting from the use of organic acid (i.e., malate and sodium acetate) or molasses. Accordingly, molasses could be a suitable substrate to replace malate as a carbon source for *R*. *palustris* PS3 cultivation. Taken together, we selected CLS and molasses as nitrogen and carbon sources for future experiments.

### Screening suitable fermentation conditions

For the purpose of screening suitable fermentation conditions, the range and levels of test variables were evaluated. We made a preliminary investigation for the concertation effects of CSL and molasses on the growth of *R*. *palustris* PS3 by OFAT (S2A and S2B Fig in S1 File). Subsequently, the effects of pH values, temperatures and dissolved oxygen (%) were respectively evaluated for PS3 growth (S2C-S2E Fig in S1 File). These derived data were used for fractional factorial design (FFD).

All of the experiments in FFD were performed in the 5 L desktop bioreactor. The factor screening process and the experimental design for the range and levels of test variables, as well as results for the FFD are shown in S2 Table in S1 File. The experimental data were analyzed by ANOVA with a first-order regression model (S1 Eq in S1 File). The results of the regression analyses are shown in S3 Table in S1 File and were analyzed by Fisher’s F-test and Student’s t-test. Student’s t-test was used to evaluate the significance of the factor regression coefficients. The proposed linear model for the biomass (g/L) of *R*. *palustris* PS3 is shown in S2 Eq in S1 File. As shown in S3 Table in S1 File, the model *p* value is significantly lower than 0.05, and the coefficient and adjusted coefficient of determination *R*^2^ were calculated to be 0.9999 and 0.9996, respectively. The coefficients of the factors are 0.76188 (CSL), 0.58188 (molasses), 0.00438 (dissolved oxygen), 0.34688 (temperature) and -0.08312 (pH), respectively (S3 Table in S1 File). In addition, for the regression *p* value, all of the target factors showed a significant effect except that of dissolved oxygen (DO). Therefore, we set the dissolved oxygen as constant at 30% for the subsequent experiments. To further identify the appropriate range of variables for RSM, we conducted the steepest ascent path method to clarify the levels of factors. The steepest ascent path was designed according to S2 Eq in S1 File., and the results are shown in S4 Table in S1 File. The search direction and length of each factor were CSL 5 mL/L, molasses 3.82 g/L, temperature 0.7°C and pH -0.3, respectively. Based on the data, we found that the biomass significantly increased with each step, reaching 2.44 g/L in step 4. After step 5, the biomass of *R*. *palustris* PS3 dramatically decreased and was even lower than the original biomass.

### Optimization of culture conditions by RSM

According to the results of the factorial design experiment (S3 Table in S1 File), we took CSL, molasses, temperature and pH as the major variables affecting the performance of *R*. *palustris* PS3 growth. We conducted a four-coded-level central composite design (CCD) to optimize the levels of these variables. The corresponding experimental results of the CCD design are shown in Table 2 and were fitted with a second–order polynomial equation (Eq 2). The proposed polynomial model for biomass production in *R*. *palustris* PS3 is shown in Eq (3):

$$\text{Biomass}\;\left(\text{g}/\text{L}\right)=4.2267-0.1918x_{1}-0.0562x_{2}-0.6582x_{3}+0.4321x_{4}-0.3882x_{1}^{2}-0.0442x_{2}^{2}-0.7315x_{3}^{2}-1.0302x_{4}^{2}+0.0165x_{1}x_{2}-0.1448x_{1}x_{3}+0.2340x_{1}x_{4}$$ Eq (3)

**Table 2:** The coded levels and real values for the experimental design and results of CCD.

Trial no.	Coded levels of factors				Biomass (g/L)	
	*x*_1_	*x*_2_	*x*_3_	*x*_4_	Experimental	Predicted
1	-1	+1	+1	-1	0.88	0.74
2	-1	-1	-1	-1	1.45	1.34
3	+1	+1	+1	+1	0.91	0.86
4	+1	-1	+1	-1	0.25	0.21
5	-1	-1	+1	+1	1.09	1.02
6	-1	+1	-1	+1	1.59	1.47
7	+1	+1	-1	-1	1.09	1.00
8	+1	-1	-1	+1	1.75	1.72
9	0	0	0	0	2.29	2.18
10	0	0	0	0	2.21	2.18
11	0	0	0	0	2.31	2.18
12	0	0	0	0	2.23	2.18
13	+1.682	0	0	0	1.43	1.44
14	-1.682	0	0	0	1.63	1.78
15	0	+1.682	0	0	1.94	2.06
16	0	-1.682	0	0	2.13	2.16
17	0	0	+1.682	0	0.47	0.54
18	0	0	-1.682	0	1.59	1.68
19	0	0	0	+1.682	1.01	1.05
20	0	0	0	-1.682	0.18	0.30

Letters: *x*_1_ = CSL (mL/L), *x*_2_ = molasses (g/L), *x*_3_ = temperature (°C) and *x*_4_ = pH value. Trial no. 1 to 8 represented fractional factorial design (FFD), trial no. 9 to 12 were referred as central point, and trial no. 13 to 20 were represented as axial points. The coefficient of determination, R2, between the predicted and experimental values was calculated to be 0.98.

The variables *x*_1_, *x*_2_, *x*_3_ and *x*_4_ represent the CSL, molasses, temperature and pH value, respectively. The results of the regression analyses were evaluated by Fisher’s *F*-test and Student’s *t*-test. As shown in Table 3, the regression coefficients and corresponding *p* values of the linear terms of CSL, temperature, pH value and interaction terms between CSL and temperature or pH value had a significant effect on *R*. *palustris* PS3 biomass production (*p* value < 0.05). However, the molasses term and other interaction terms were not significant at the 5% level. Moreover, other interaction terms between molasses, temperature and pH value were omitted from the predicted model. The coefficient and adjusted coefficient of determination, *R*^2^, for the regression model fit were calculated to be 0.9962 and 0.9896, respectively. This means that more than 98% of the variability of the regression model could be explained by the regression equation. The response-surface full quadratic model of biomass for *R*. *palustris* PS3 was also tested by ANOVA. The *F* value of the model was 151.99, and the *p* value was less than 0.0001 (2.84×10^−7^), indicating that the model was highly significant (Table 3). The lack-of-fit *F* value and *p* value were 3.16 and 0.186, respectively, which implied that the lack of fit was insignificant. These results supported that the second-order model adequately approximated the response surface of *R*. *palustris* PS3 biomass production. After canonical transformation of Eq (3), the optimum fermentation combination was obtained as follows: CSL, 39.41 mL/L; molasses, 32.35 g/L, temperature, 37.9°C, pH 7.0 and the DO was constant at 30%. The model predicted that the maximum response of *R*. *palustris* PS3 biomass production would be 2.31 g/L.

**Table 3:** The variance analysis of the second-order regression model for biomass production.

Source	DF	SS	MS	*F*-value	Coef.	*p*-value
Model	12	31.9509	2.6626	151.99		2.84E-07
Blocks	1	0.4613	0.4613	26.33		0.0003
Linear	4	9.0117	2.2529	128.6		1.2E-06
*x*_1_	1	0.5024	0.5024	28.68	-0.1918	0.00104
*x*_2_	1	0.0432	0.0432	2.46	-0.0562	0.15911
*x*_3_	1	5.9164	5.9164	337.72	-0.6582	2.8E-07
*x*_4_	1	2.5498	2.5498	145.55	0.4321	6E-06
Square	4	21.5663	5.3916	307.76		5.9E-08
$x_{1}^{2}$	1	2.143	2.143	122.33	-0.3882	1E-05
$x_{2}^{2}$	1	0.0278	0.0278	1.59	-0.0442	0.23298
$x_{3}^{2}$	1	7.6097	7.6097	434.38	-0.7315	1.4E-07
$x_{4}^{2}$	1	15.0951	15.0951	861.66	-1.0302	1.3E-08
2-Way Interaction	3	0.608	0.2027	11.57		0.00412
*x*_1_*x*_2_	1	0.0022	0.0022	0.12	0.0165	0.73435
*x*_1_*x*_3_	1	0.1677	0.1677	9.57	-0.1448	0.0172
*x*_1_*x*_4_	1	0.4382	0.4382	25.01	0.234	0.00153
Error	7	0.1226	0.0175			
Lack-of-Fit	4	0.0991	0.0248	3.16		0.186
Pure Error	3	0.0235	0.0078			
Total	19	32.0735				

Letters: *x*_1_ = CSL (mL/L), *x*_2_ = molasses (g/L), *x*_3_ = temperature (°C) and *x*_4_ = pH value.

### Effects of various factors on *R*. *palustris* PS3 biomass production

**Fig 2:**
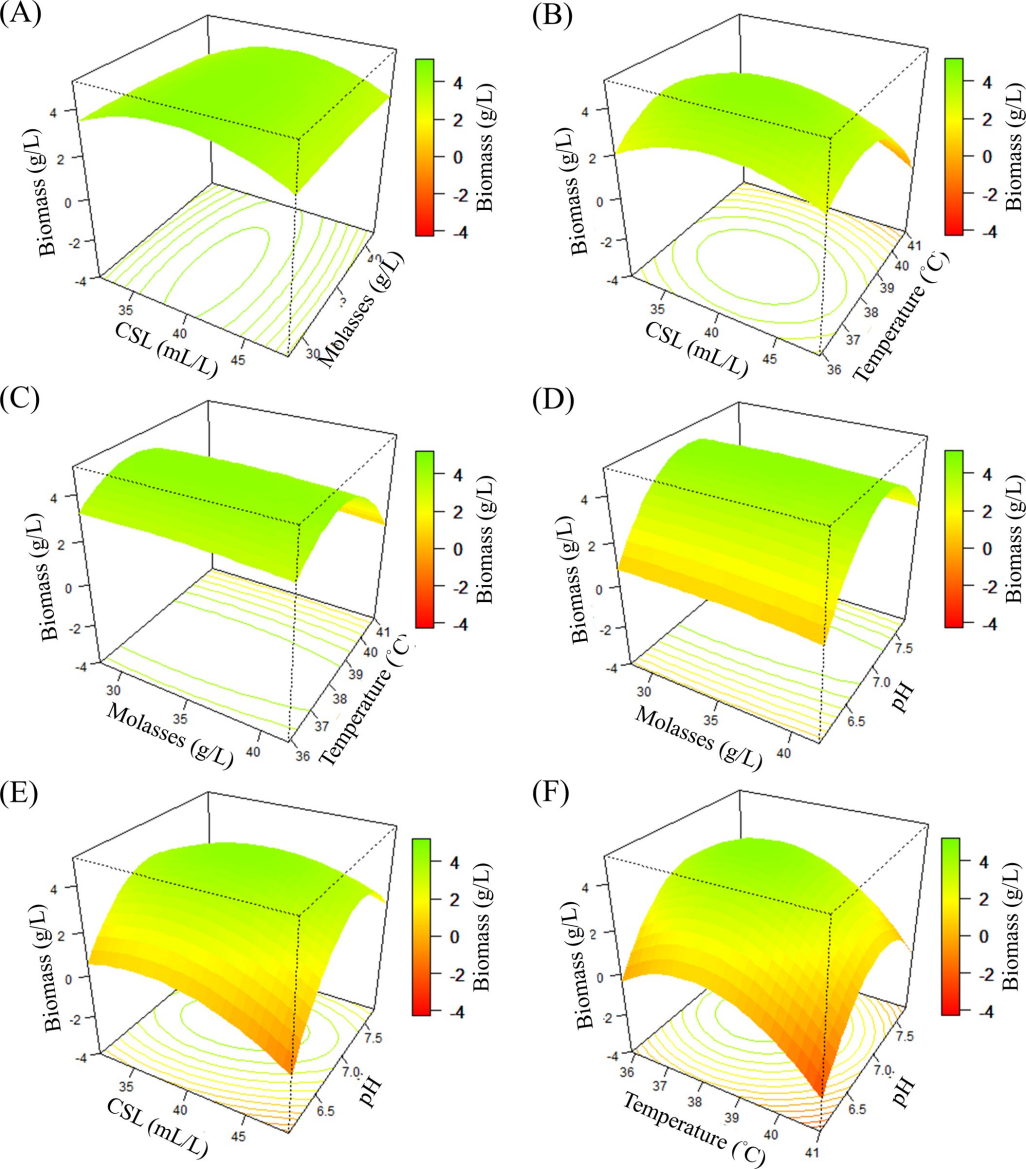
Three-dimensional response surfaces and contour plots of the effects of three factors on *R*. *palustris* PS3 biomass production. When two factors were plotted, the other two factors were set at the coded level, zero. Each condition was as follows: CSL, 40 mL/L; molasses, 35 g/L; temperature, 38.5°C and pH value, 6.9. (A): CSL and molasses were plotted at 38.5°C and pH 6.9; (B) CSL and temperature were plotted at 35 g/L molasses and pH 6.9; (C) molasses and temperature were plotted at 40 mL/L CSL and pH 6.9; (D) molasses and pH were plotted at 40 mL/L CSL and 38.5°C; (E): CSL and pH were plotted at 35 g/L molasses and 38.5°C; (F): temperature and pH were plotted at 40 mL/L and 35 g/L molasses. CSL: corn steep liquor.

The 3D response surface contour plots were constructed in R software to analyze the interaction effects of four variables (CSL, molasses, temperature and pH value). The plots showed the effect of two variables on the response while the other factors were set at the “zero” level; the levels tested were 40 mL/L CSL, 35 g/L molasses, 38.5°C temperature and 6.9 pH (Fig 2). These contour plots represent the projection maps the 3D response surfaces onto two-dimensions planes with contours delineating changes in 3D space, which provide relative clear pictures of the responses derived from each factor. As shown in Fig 2A and 2B and 2E, the elliptical and inclined form of contour plots inferred that the interactions between CSL and molasses, CSL and temperature, CSL and pH value are evident. These results can also be confirmed by the variance analysis of regression model for biomass production (Table 3). On the other hand, the biomass production of *R*. *palustris* PS3 significantly increased with increasing CSL, temperature and pH. However, too high a concentration or condition level of these factors resulted in the opposite effect. On the other hand, it was noted that molasses did not acutely affect the biomass production of *R*. *palustris* PS3 under our experimental design, although increasing the concentration resulted in a slight increase-then-decrease pattern. These results were consistent with the polynomial regression analyses (Table 3), i.e., biomass production was significantly influenced by CSL, temperature and pH value. On the other hand, the 3D surface projection was confined to the smallest curve of the contour diagram suggesting that it contained an optimum condition in the levels of variables. Moreover, the 3D response surface presented a “roof form”. Taken together, these data suggest that the model has a maximum stationary point, which contains the maximum biomass production of *R*. *palustris* PS3 strain.

### Verification of optimization

To verify the predicted biomass production of *R*. *palustris* PS3, confirmation fermentation with the predicted optimal culture conditions (CSL, 39.41 mL/L; molasses, 32.35 g/L, temperature, 37.9°C, pH 7.0 and DO 30%) was performed. As shown in Table 4, after 24 hours of fermentation, the biomass production of *R*. *palustris* PS3 was 2.18 ± 0.01 g/L, which was approximately 7.8 times higher than that obtained in the original PNSB medium (0.28 ± 0.01g/L). The validated biomass production showed a high correlation (95%) with the predicted biomass from the response model. This result suggests that the proposed model Eq (3) is effective for *R*. *palustris* PS3 biomass production. The estimated cost of this newly developed medium was about 0.16 US$/L, which was approximately 30% of the original PNSB medium (0.53 US$/L). The detailed material cost of each component in respective medium was shown in S5 Table in S1 File.

**Table 4:** Comparison of PNSB medium and optimal medium parameters for *R*. *palustris* PS3 biomass production for 24 hours.

Factor	PNSB medium	Optimal medium
Molasses (g/L)	-	32.35
Malate (g/L)	5	-
CSL (mL/L)	-	39.41
Yeast extract (g/L)	1	-
NH_4_Cl (g/L)	0.5	-
Agitation (rpm)	200	-
Aeration (vvm)	1.22	-
pH	7	7
Temperature (°C)	37	37.9
Biomass (g/L)	0.28 ± 0.01	2.18 ± 0.01
Material cost (US$/L)	0.53	0.16

The fermentation experiments were carried out in a 5-L bioreactor.

Material cost was calculated according to S5 Table in S1 File, which shown detailed cost information for each component in respective medium.

### Validation of the plant growth-promoting effect of the newly developed fermentation broth

**Fig 3:**
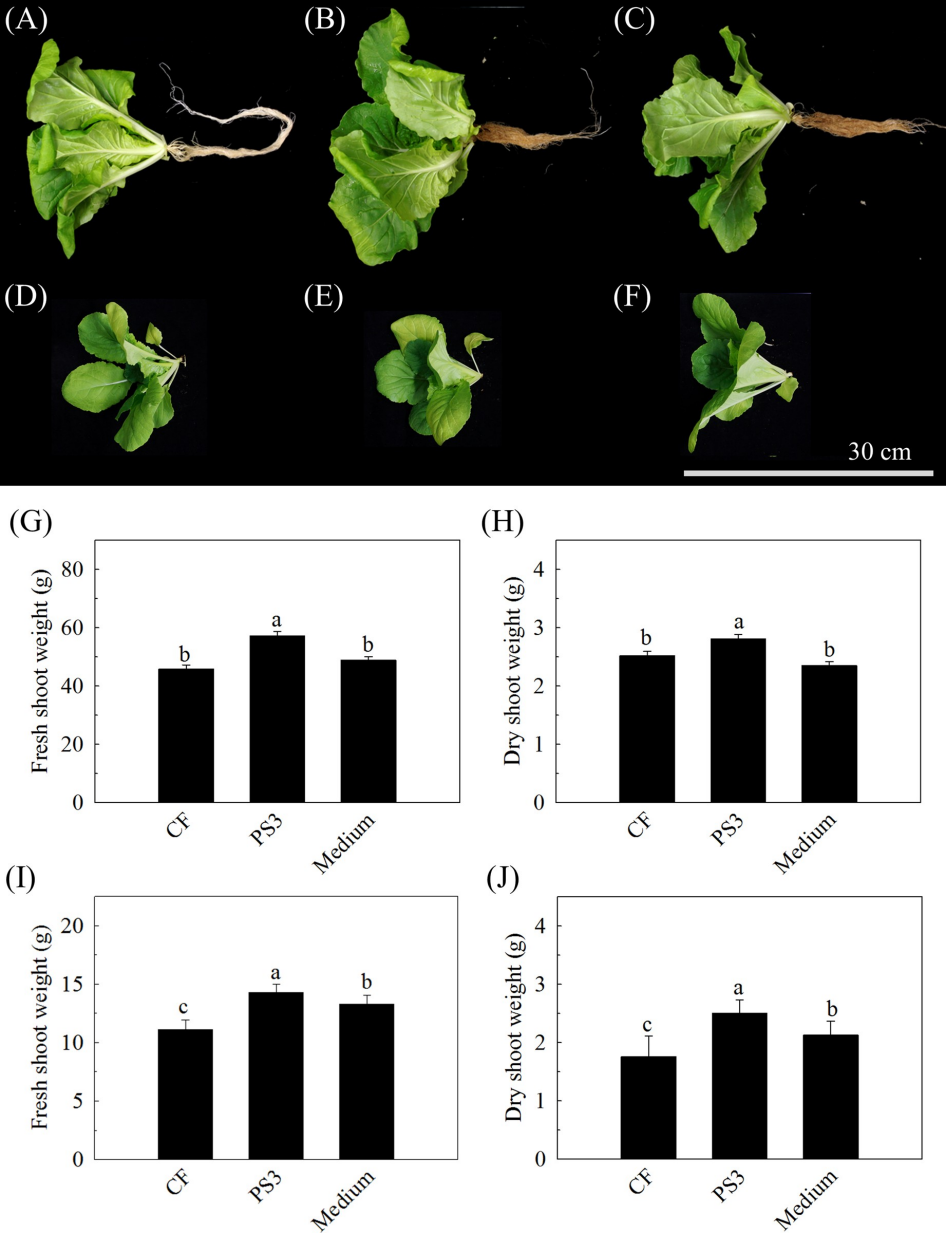
Plant growth-promoting effects of *R*. *palustris* PS3 incubated with the newly developed culture conditions on leafy vegetable. *Brassica rapa chinensis* (Chinese cabbage) was cultivated with pure chemical fertilizer under either hydroponic or soil system. “CF” indicates the control group (chemical fertilizer treatment), “PS3” indicates the treatment (CF) supplemented with the fermentation broth of *R*. *palustris* PS3, “medium” indicates the treatment (CF) supplemented with fresh medium, respectively. (A)-(F) show the morphology of Chinese cabbage cultivated in hydroponic (A:CF, B: PS3 and C: medium) and soil systems (D: CF, E: PS3 and F: medium) with the different treatments at 17 DAP and 28 DAP, respectively. (G-J) are the fresh and dry shoot weights of Chinese cabbage cultivated in hydroponic (G and H) and soil systems (I and J), respectively. Vertical bars represent the standard error and bars with different letters indicate statistically significant differences at P = 0.05 according LSD test.

To confirm the plant growth-promoting effect of PS3 with the newly developed fermentation broth, we cultivated Chinese cabbage in either hydroponic or soil system with different treatments. As shown in S6 Table in S1 File, the fermented culture broth of PS3 contained 0.05±0.17 g/Kg of total organic carbon and 16.98 ±0.17 g/Kg of total nitrogen (S6 Table in S1 File), indicating the C/N ratio of this broth was approximated 1.77. The morphologies of 17 DAP and 28 DAP Chinese cabbage cultivated under different treatments are shown in Fig 3A–3F. In the hydroponic system, the Chinese cabbage treated with the PS3 inoculant was obviously larger than that treated with CF or conventional growth medium. There was no significant difference in the size of Chinese cabbage treated with the medium and with CF. The fresh and dry shoot weights of Chinese cabbage cultivated in the hydroponic system with different treatments are shown in Fig 3G–3H (fresh/dry weight of CF: 45.87 ± 1.39 g/2.51 ± 0.076 g, PS3: 57.20 ± 1.54 g/2.81 ± 0.078 g, medium: 48.86 ± 1.14 g/2.35 ± 0.067 g). Compared to those of the CF group, the fresh and dry shoot weights of the PS3 treatment increased by 25% and 12%, respectively. Likewise, the fresh and dry shoot weights of PS3 Chinese cabbage cultivated in the soil system were also superior to those of the other treatments (fresh/dry weight of CF: 11.11 ± 0.84 g/1.75 ± 0.36 g, PS3: 14.27 ± 0.71 g/2.50 ± 0.23 g, medium: 13.31 ± 0.71 g/2.12 ± 0.24 g, respectively) (Fig 3I and 3J). Compared to those of the CF group, the fresh and dry shoot weights were 28% and 48% increased, respectively, with the PS3 treatment. We confirmed that the fermentation broth of *R*. *palustris* PS3 produced under the newly developed culture conditions had beneficial effects on plant growth.

## Discussion

*R*. *palustris* PS3 has been shown to have beneficial effects on several crops [8, 11, 32]. To scale up the biomass production of PS3 for commercial purposes, we developed optimal fermentation conditions that are cost-effective and have a high product yield and a short fermentation time. Based on the derived RSM model (Eq (3)), the predicted optimal culture conditions were 39.41 mL/L CSL, 32.35 g/L molasses, 37.9°C and 7.0 pH. As shown in Table 4, the optimized medium resulted in *R*. *palustris* PS3 biomass production (2.18 ± 0.01 g/L at 24 hours fermentation) that was 7.8-fold higher than that with the original PNSB medium (0.28 ± 0.01g/L at 24 hours fermentation). Accordingly, this optimal fermentation condition for *R*. *palustris* PS3 is not only highly productive but also cost- and time-effective. It has been indicated that the sources of nitrogen and carbon play a critical role in the production of microbial secondary metabolites [37, 38]. Variation in fermentation condition results in changes in the yields and compositions of these secondary metabolites, which potentially affect activity, biomass and original effectiveness of microorganisms [39–41]. Therefore, we deduced that the secondary metabolites of PS3 using CLS and molasses as nitrogen and carbon sources can effectively stimulate the growth of this bacterium, although the profiles of the substances remains to be elucidated.

For industrial fermentation, the composition of the medium is critical, since it significantly affects the product concentration, yield and volumetric productivity. Furthermore, the cost of raw materials can range from 40% to 80% of the total costs of fermentation and affect the ease and cost of downstream product separation [42]. *R*. *palustris* is already known as the most metabolically versatile bacteria, which was able to catabolize various carbon and nitrogen sources, such as glucose, fructose, malic acid, acetic acid, ammonium nitrate, glutamine, yeast extract and so on [8, 26, 43, 44]. In this study, we evaluated several low-cost materials derived from common nutrients or agro-industrial byproducts for the fermentation of *R*. *palustris* PS3 and found that a variety of nitrogen and carbon sources could be utilized (Fig 1). These results may be attributed to the extraordinary metabolic versatility of *R*. *palustris* PS3 [45]. Noteworthily, the growth rates of *R*. *palustris* PS3 varied in response to different carbon or nitrogen sources (Fig 1). For nitrogen sources, the viable count of PS3 cultured with complex substrates, such as CSL, BRE or yeast extract supplied with NH_4_Cl (i.e., the PNSB treatment), was significantly higher than that of PS3 cultured with a sole nitrogen source (i.e., NH_4_NO_3_) (Fig 1B). These complex substrates all contain nitrogen-rich substances [46–49], and were well solubilized in our cultivation medium. On the other hand, although SF and SPI are also complex nitrogen sources, the cell counts of these two treatments were relatively low (Fig 1B). We deduced that it was due to their low solubility in the medium as described above or because their primary components (β-conglycinin, glycinin, and lipophilic proteins) are not easily metabolized by *R*. *palustris* PS3. However, further experimentation is still needed.

For carbon sources, as shown in Fig 1D, the viable count of PS3 cultured with molasses was higher than those of PS3 cultured with sugars (i.e., glucose or fructose). Molasses is a by-product of the sugar manufacturing process and may be obtained from beet or sugarcane; it contains abundant saccharides (such as sucrose, glucose, and fructose) and small amounts of organic acids (such as acetic acid and lactic acid) [50, 51]. It has been reported that *R*. *palustris* biomass production is stimulated by the co-utilization of multiple carbon sources [52]. Similar phenomenon was also observed in other microorganisms. For example, the growth rate of cultivated *E*. *coli* was higher with a combination of two substrates (i.e., mannose, xylose, glycerol, maltose or glucose supplemented with succinate, pyruvate, oxaloacetate, glycerol or glucose, respectively) than with a single substrate (mannose, xylose, glycerol, maltose or glucose, respectively) [53]. *Lactobacillus brevis* subsp. *lindneri* CB1 cultivated with maltose and citrate mixtures resulted in a faster cell growth rate than that of the strain cultivated with maltose alone, and the growth rate and viable counts (OD_620_) derived from the former media were 1.8-fold and 1.2-fold higher, respectively [54]. Comparison with either glucose alone or malate alone as a carbon source, the growth rate of *B*. *subtilis* 168 increased 1.25-fold when cultured in medium containing glucose and malate mixtures [55]. It is already known that *R*. *palustris* can readily utilize organic acids as carbon sources [56]. Since organic acids such as acetic acid and lactic acid are contained in molasses [51], it is possible that *R*. *palustris* co-metabolized several of the carbon sources in molasses, although further experimentation is needed.

The relationship between the target factors and *R*. *palustris* PS3 biomass production was explained by mathematical models and ANOVA. The linear regression model and ANOVA in FFD suggested that increasing the concentration of molasses could increase biomass production (S2 Eq and S3 Table in S1 File). This result is quite reasonable because bacterial cells require carbon sources to survive. However, the results of the second-order regression model and ANOVA (Table 3) indicated that molasses did not significantly influence the growth of *R*. *palustris* PS3 (Eq (3) and Table 3). We deduced that this discrepancy between the first- and second-order regression models might be attributed to CSL, which contains organic acids [48]. Increasing the concentration of CSL might supply a carbon source to partially substitute for molasses. In a previous study, it was mentioned that CSL could serve as a supplement to replace carbon sources for some microorganisms [57]. It also indicated that the carbon source was already sufficient in our CCD experiments. The effects of molasses concentration can also be observed in Fig 2A, 2C and 2D. Increasing or reducing the concentration of molasses did not significantly alter the biomass of *R*. *palustris* PS3.

In contrast, the factors CSL, temperature and pH value significantly influenced the growth of *R*. *palustris* PS3 (Table 3). It was observed that for greater and lower levels of CSL, temperature and pH value, there was a reduction in the response (Fig 2). Not surprisingly, a higher or lower pH value reduced the growth of *R*. *palustris* PS3, while the optimal pH value proposed by the RSM model for the fermentation of *R*. *palustris* was 7.0 (Table 4). This result was consistent with our previous study, which found that the pH range for *R*. *palustris* PS3 growth was 5.0 to 9.0, and the optimum pH was 7.0 [8]. Interestingly, the mathematical model suggested that the optimal temperature for *R*. *palustris* PS3 fermentation was 37.9°C, which was different from our previous finding (30°C) [8]. We inferred that this dissimilarity could be attributed to the culture medium composition. Our proposed model showed that the interaction between CSL and temperature dramatically influenced *R*. *palustris* PS3 biomass production (Table 3). However, no study has indicated the effects of temperature and nutrients on *R*. *palustris* metabolism. Likewise, a higher temperature suppressed the growth of *R*. *palustris* PS3, though S2 Eq in S1 File indicated that increasing the temperature could increase *R*. *palustris* PS3 biomass production (Fig 2B, 2C and 2F). In the CCD experiments, the biomass production in trials No. 1, 3, 4, 5 and 17 was significantly lower than that in the other trials (Table 2). Regarding the effect of CSL on the growth of *R*. *palustris* PS3, we considered that an extra nitrogen source was absolutely important to *R*. *palustris* growth. It is known that *R*. *palustris* can obtain nitrogen from air by biological nitrogen fixation (BNF) [1]. However, BNF is very sensitive to the O_2_ concentration and is only carried out under microaerobic conditions. Thus, under the aerobic culture conditions of this study, BNF did not occur. Accordingly, supplementation with exogenous nitrogen in the culture medium was necessary.

Molasses and CSL can be applied individually as fertilizers in agriculture. For example, it has been found that the soil quality was remarkably improved by the application of molasses [58, 59]. There were also reports showing that molasses could increase the yield and quality of crops [60, 61]. CSL was shown to have beneficial effects on plants [62, 63], and supplementation of with exogenous CSL in soil promoted the growth of the root system of soybean [62]. It has been suggested that the organic nitrogen in CSL is converted to nitrate via microbial ammonification and nitrification and directly utilized by plants [64]. However, it has also been reported that CSL and molasses might have negative effects on plant growth. Zhu et al [62] reported that high concentration of CSL (more than 2%) will inhibit plant growth. In addition, fertilization of molasses singly or in combination with some PGPRs (*Bacillus* spp. *Azospirillum* spp. or *Azotobacter* spp.) showed a negative influence on seed germination *in vitro* [65]. In this study, the newly developed medium containing molasses (32.35 g/L) and CSL (39.41 mL/L) was verified for its effect on plant growth. Since the PS3 fermentation broth showed positive effects on plant growth in both soil and hydroponic systems (Fig 3). It suggests that this medium is suitable not only for phototrophic bacteria production but for application in agricultural production. Such an approach to farming is regarded as environmentally friendly and can be used to reduce excessive chemical fertilizer application and ensure sustainable crop production.

Furthermore, it is notable that PS3 fermentation broth obtained from newly developed medium that is consistent with PS3 cultured in modified PNSB, it shown the plant growth promotion effect (Fig 3). In our previous work [8], to examine whether the plant beneficial effects of *R*. *palustris* PS3 were elicited by viable cells or conferred by organic compounds from the PNSB medium or dead/ decaying cells, the 65°C heat-killed bacterial suspension was applied to replace the vegetative *R*. *palustris* cells. The results showed that neither medium nor dead *R*. *palustris* cells was able to promote plant growth [8]. These results indicated that the effectiveness observed were mainly exerted by the viable cells of *R*. *palustris* PS3. Notably, it was also demonstrated that not only the population (i.e viability) but also the metabolic activity (i.e vitality) of PS3 cells is crucial for the plant beneficial traits [32]). On the other hand, in our another study [45], we carried out a comparative analysis of effective (strain PS3) as well as ineffective (strain YSC3) *R*. *palustris* strains in plant-growth promotion. PS3 and YSC3 exhibited a very close phylogenetic relationship and shared several conserved regions and genetic arrangements in their chromosomes. Although these strains have many plant growth-promoting (PGP) genes in common, only PS3 exhibited beneficial traits. We noticed that the transcripts of genes associated with bacterial colonization and biofilm formation in response to root exudates were higher in PS3 than those in YSC3 strain [45]. These data suggested that PS3 responds better to the presence of plant hosts. It indicates that the physiological responses of this bacterium to its plant hosts as well as successful establishment of interactions with plant hosts appear to be critical factors for PS3 to promote plant growth. Taken together, we deduced that the beneficial effects of PS3 were mainly offered by the viable cells of this bacterium through interactions with the host.

## Conclusion

This paper presented an experimental design for the optimization of *R*. *palustris* PS3 biomass production with an alternative, low-cost medium containing agricultural byproducts. CSL and molasses were identified as potential nitrogen and carbon sources for *R*. *palustris* fermentation. The utilization of CSL and molasses as raw materials for *R*. *palustris* fermentation can aid in reducing agro-industrial waste. The response surface methodology revealed the factors that greatly influence *R*. *palustris* PS3 growth, namely, CLS, temperature and pH value. *R*. *palustris* PS3 biomass production increased significantly, by 7.8-fold, from 0.28 ± 0.01 g/L to 2.18 ± 0.01 g/L, compared to that under the basal medium/conditions when the strain was cultivated in the optimal culture conditions (CSL, 39.41 mL/L; molasses, 32.35 g/L; temperature, 37.9°C; pH, 7.0 and DO 30%) developed by statistical experimental methods. The in planta experiments verified that the newly developed fermentation broth retained the plant growth-promoting functions of *R*. *palustris* PS3. Compared with those in previous studies, our newly developed fermentation process could successfully produce high levels of *R*. *palustris* in a shorter time. This study described the prospective uses of agro-industrial techniques for *R*. *palustris* biofertilizer production.

## Supporting information

S1 File (DOCX)
