# Supplementary material for: Development of a low-cost culture medium for the rapid production of plant growth-promoting Rhodopseudomonas palustris strain PS3
Source: PLoS One. 2020 Jul 30;15(7):e0236739. doi: 10.1371/journal.pone.0236739 (PMC7392278; doi:10.1371/journal.pone.0236739)
Supplement: S1 File — (DOCX) [file pone.0236739.s001.docx]

**Supplementary Materials and Methods**

**Development of a low-cost culture medium** **for the rapid production of plant growth-promoting *Rhodopseudomonas palustris***

**Running title: Optimization of culture conditions for *Rhodopseudomonas palutris* with agro-industrial by-products**

**Kai-Jiun Lo**^1^**, Sook-Kuan Lee**^1^ **and Chi-Te Liu**^1, 2*^

^1^ Institute of Biotechnology, National Taiwan University, R412, No. 81, Chang-Xing St, Taipei 106, Taiwan

^2^ Agricultural Biotechnology Research Center, Academia Sinica, No. 128 Sec. 2, Academia Rd., Nankang, Taipei 115, Taiwan

*** Correspondence: Chi-Te Liu**

E-mail: [chiteliu@ntu.edu.tw](mailto:chiteliu@ntu.edu.tw)

**Screening suitable fermentation conditions**

For the purpose of screening suitable fermentation conditions, the range and levels of test variables (CSL, molasses, dissolved oxygen, temperature and pH value) were preliminarily evaluated by OFAT. The influence of CSL concentrations (10, 20, 30 and 40 mL/L in medium) on the growth of PS3 was examined in the presence of molasses (5 g/L), and cultured at 37°C. The effect of molasses concentrations (0, 5, 10, 20 and 30 g/L) on the PS3 biomass production was examined in the presence of CSL (20 mL/L) in the medium, and cultured at 37°C. The effect of temperatures on the growth of PS3 was investigated by incubating in the medium containing 20 CSL (mL/L) and molasses (5 g/L) at 28°C, 32°C, 37°C and 42°C, respectively. All above experiments were performed in 250 mL Erlenmeyer flask containing 50 mL medium and then cultured with shaking at 200 rpm in the dark.

Follow-up experiments were carried out in a 5-L stirred tank bioreactor (BTF-A5L, BIOTOP Inc, Taiwan). The effect of pH value was investigated by incubating at pH 6, pH 7 and pH 8, respectively. CSL (20 mL/L) and molasses (5 g/L) were used as nitrogen and carbon sources in the medium, respectively. Aeration and agitation rate were set at 1.0 vvm and 200 rpm, respectively. The influence of dissolved oxygen was evaluated by incubating in the medium containing 20 CSL (mL/L) and molasses (5 g/L) with 20%, 40% and 60% of dissolved oxygen, respectively. The temperature and pH value were set at 37°C and 7.0, respectively.

**Evaluate the effects of fermentation factors on the growth of *R. palustris* by fractional factorial design (FFD)**

The effects of two medium components (carbon and nitrogen) and three fermentation conditions (dissolved oxygen (DO), temperature and pH) on *R. palustris* PS3 biomass production were investigated with a two-level fractional factorial design (FFD). The 2^5-2^ FFD was performed in R software with the package “rsm” and resulted in a total of 8 experiments ^(Lenth, 2009)^. The variable levels, coded variables, experimental design and results are shown in Table S1. Here, $x_{1}$,$x_{2}$,$x_{3}$,$x_{4}$ and $x_{5}$ represent CSL, molasses, DO, temperature and pH, respectively. All of the experimental data for the FFD were fitted with a standard first-order equation by a multiple regression technique. The regression equation is shown as follows:

Standard first-order regression equation, S1 Eq.:

$Y=\beta_{0}+\sum_{i=1}^{k} \beta_{i}x_{i}$ S1 Eq.

where $Y$ is the predicted response of the biomass (g/L) of *R. palustris*, $\beta_{0}$ is the intercept, and$\beta_{i}$ and $x_{i}$ are the linear constant coefficients and coded independent variables for factors, respectively. The quality of fit of the first-order equation model to the data was analyzed by the coefficient of determination, R-squared, and ANOVA. The statistical software used was R, version 3.6.2 (R Core Team, 2019). All experiments were performed in a bioreactor.

**Maximum region improvement of factors by steepest ascent method**

To maximize the region of each factor and achieve a maximum for the response of interest, the path of steepest ascent method was applied in this study. The search direction and length of the step were estimated according to the ratio of coefficients in S2 Eq., and adjusted from the central operating conditions in FFD. S2 Eq. was generated by data collected from FFD and fitted with first-order regression equation S1 Eq (1).. The search direction and length of each factor were CSL 5 mL/L, molasses 3.82 g/L, temperature 0.7°C and pH -0.3, respectively. The experimental design is shown in Table S3. All experiments were performed in a bioreactor and fermentation was performed in 24 hours.

**Quantification of total organic carbon**

The total organic carbon in fermentation broth was quantified by Walkley-Black chromic acid wet oxidation method (Nelson and Sommers, 1982) with some modification. Weighed 5.0 g of fermentation broth into a 500 mL erlenmeyer flask. Add 10 mL of 0.1N K_2_Cr_2_O_7_ and swirl the flask gently to disperse the sample in the solution. Rapidly, 20 mL of concentrated H_2_SO_4_ was added into above mix solution. Subsequently, the flask was stood on an insulated sheet for 30 min in a fume hood. After that, 200 mL of D.D. water was added into the flask and mix with 10 mL of 85% H_3_PO_4_. Finally, 30 drops of barium diphenylamine sulfonate indicator were added into above mix solution and titrated the solution with 0.5 M (NH_4_)_2_ Fe(SO_4_)_2_•6H_2_O. At the end-point of titration, the color changes sharply to brilliant green. The total organic carbon (g/Kg) was calculated by equation as below:

$$Total organic carbon \left( g/{Kg} \right)=V\times\left( 1-\frac{V_{s}}{V_{b}} \right)\times\frac{12}{4\times1000}\times1.3\times\frac{1000}{sample \left( g \right)}$$

Where, the *V* is the volume of 0.1N K_2_Cr_2_O_7_ (mL), *V_s_* is the volume of titrant in 0.5 M (NH_4_)_2_ Fe(SO_4_)_2_•6H_2_O (mL) and *V_s_* is the volume of titrant in blank, D.D. water (mL).

**Quantification of total nitrogen**

Quantification of total nitrogen was carried out by Kjeldahl method (Bremner and Mulvaney, 1982) with some modification. Weighted 1.0 g fermentation broth and mix with 1.g catalyst, which is consists of K_2_SO_4_, CuSO_4_•5H_2_O and Selenium (100:10:1 in weight). After that, 10 mL concentrated sulfuric acid was added. The above mixture was heated for 2 hours. After cooling, the mixture was filtered to obtain the clear supernatant. The above 10 mL solution was mixed with 5 mL 10N NaOH and 0.2 g Devarda's Alloy, then this mixture was heated to liberate ammonia which is distilled by steam through a condenser, the tip of which is submerged in a flask containing 10 mL 2% boric acid containing Tashiro indicator (2 volume of 0.2% methyl red in 90% ethanol + 1 volume of 0.2% methylene blue in 90% ethanol). After the distillation was finished, titrate the ammonia with 0.1 N HCl. The total nitrogen was calculated according to equation as shown below:

$$Total nitrogen \left( g/{Kg} \right)=\frac{(V_{s}-V_{b})\times0.09780\times14\times V_{tse}}{W_{s}\times V_{se}\times recorver rate}$$

Where, *V_s_* is the volume of titrant in 0.01N HCl, *V_b_* is the volume of titrant in blank (D.D. water), *V_tse_* is the volume of total sample extraction solution (one mL in this study), *W_s_* is the weight of sample and *V_se_* is the volume of reaction sample extraction (one mL in this study). Recover rate was calculated according equation as shown in below, and 10 N NH_4_Cl was used as standard reagent.

$$Recovery rate \left( \% \right)=\frac{\left( V_{std}-V_{b} \right)\times0.09780\times14\times100\%}{C_{std}\times\frac{V_{STD}}{1000}}$$

Where, *V_std_* is the volume of titrant in 10 N NH_4_Cl, *V_b_* is the volume of titrant in blank (D.D. water), 0.09789 is the standard equivalent concentration of 0.1 N HCl which was calibrated by titration with 0.1 N NaOH. *C_std_* is the equivalent concentration of NH_4_Cl (10 N) and *V_STD_* is the volume (10 mL).

**S2 Eq.:**

$Biomass (g/L)=1.36149+0.393x_{1}+0.29241x_{4}+0.00226x_{3}+0.17893x_{4}-0.04288x_{5}$

**Supplementary Figures**

**S1 Fig. The biomass production of *R. palustirs* PS3 cultured in the medium containing molasses and corn steep liquor for 24 hours.** *R. palustirs* PS3 was incubated in 250 mL flask containing PNSB medium under 200 rpm rotation at 37°C. The 5 g/L molasses and 1.5 mL/L corn steep liquor were substituted for malate, yeast extract CFU/mL. The circular symbols present the OD_600_ and triangle symbols indicate the CFU/mL.

**
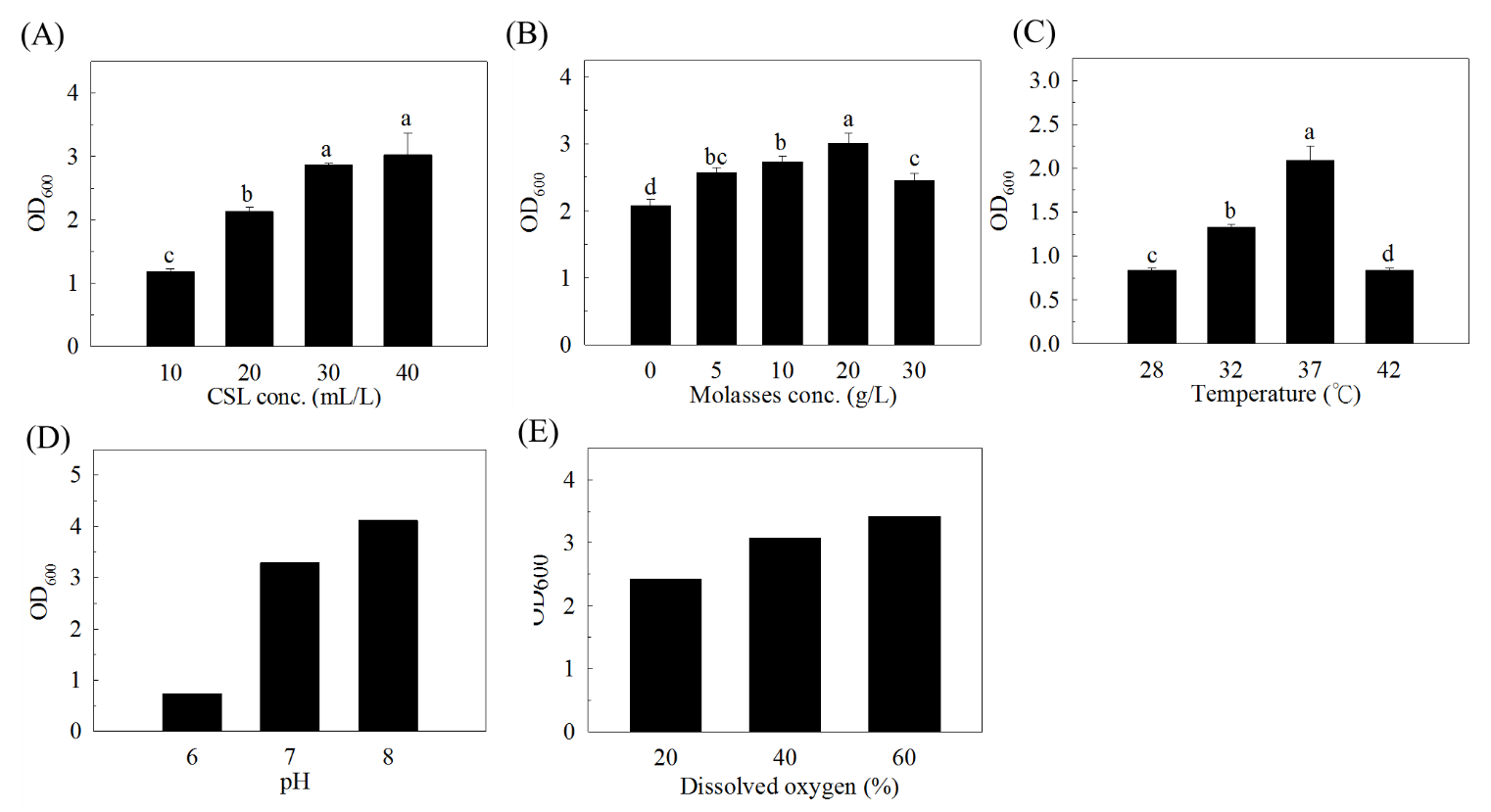
**

**S2 Fig. The effects of different culture conditions on the growth of *R. palustris* PS3 strains. All of the experiment were performed within 24 hours.** The experiments (a), (b) and (c) were performed by flask culture with 200 rpm under dark. The experiments (d) and (e) were carried out in 5-L bioreactor. The aeration in the experiment (d) was set at 1.0 vvm and the agitation was set in 200rpm. The aeration in experiment (e) was constant at 1.0 vvm, and the agitation was regulated by computer. (a) *R. palustris* PS3 was incubated under different concentrations of CSL at 37°C, and 5 g/L molasses was used as alternative carbon source in the medium. (b) *R. palustris* PS3 was incubated in different concentrations of molasses at 37°C, and 20 mL/L CSL was used as alternative nitrogen source in the medium. (c) *R. palustris* PS3 was incubated at different temperatures (°C), and 20 mL/L CSL and 5 g/L molasses were used as nitrogen and carbon sources, respectively. (d) *R. palustris* PS3 was incubated at different constant pH values at 37°C, and 20 mL/L and 5 g/L molasses were used as nitrogen and carbon sources, respectively. (e) *R. palustris* PS3 was incubated at various dissolved oxygen (%) at 37°C, and 20 mL/L and 5 g/L molasses were used as nitrogen and carbon sources, respectively.

**Supplementary Tables**

| **S1 Table. The coded levels and real values for the experimental design for CCD.** | | | | |
| --- | --- | --- | --- | --- |
| Trial no. | Coded levels of factors | | | |
|  | $x_{1}$ | $x_{2}$ | $x_{3}$ | $x_{4}$ |
| 1 | -1 | +1 | +1 | -1 |
| 2 | -1 | -1 | -1 | -1 |
| 3 | +1 | +1 | +1 | +1 |
| 4 | +1 | -1 | +1 | -1 |
| 5 | -1 | -1 | +1 | +1 |
| 6 | -1 | +1 | -1 | +1 |
| 7 | +1 | +1 | -1 | -1 |
| 8 | +1 | -1 | -1 | +1 |
| 9 | 0 | 0 | 0 | 0 |
| 10 | 0 | 0 | 0 | 0 |
| 11 | 0 | 0 | 0 | 0 |
| 12 | 0 | 0 | 0 | 0 |
| 13 | +1.682 | 0 | 0 | 0 |
| 14 | -1.682 | 0 | 0 | 0 |
| 15 | 0 | +1.682 | 0 | 0 |
| 16 | 0 | -1.682 | 0 | 0 |
| 17 | 0 | 0 | +1.682 | 0 |
| 18 | 0 | 0 | -1.682 | 0 |
| 19 | 0 | 0 | 0 | +1.682 |
| 20 | 0 | 0 | 0 | -1.682 |

Letters: $x_{1}$= CSL (mL/L), $x_{2}$= molasses (g/L), $x_{3}$= temperature (°C) and $x_{4}$=pH value. The trail no. 1 to 8 represented fractional factorial design (FFD); the trial no. 9, 10, 11 and 12 were referred as central point, respectively; the trail no 13 to 20 were represented as axial points.

| **S2 Table. Level codes of the variables and experimental design results from the fractional factorial design.** | | | | | | |
| --- | --- | --- | --- | --- | --- | --- |
| Trial No. | Coded levels of factors | | | | | Biomass (g/L) |
|  | $x_{1}$ | $x_{2}$ | $x_{3}$ | $x_{4}$ | $x_{5}$ |  |
| 1 | -1(10) | +1(30) | -1(10) | -1(34) | +1(7.5) | 1.03 |
| 2 | +1(30) | +1(30) | -1(10) | +1(40) | -1(6.5) | 2.3 |
| 3 | -1(10) | +1(30) | +1(50) | -1(34) | -1(6.5) | 1.13 |
| 4 | -1(10) | -1(10) | +1(50) | +1(40) | -1(6.5) | 0.89 |
| 5 | +1(30) | -1(10) | -1(10) | -1(34) | -1(6.5) | 1.32 |
| 6 | -1(10) | -1(10) | -1(10) | +1(40) | +1(7.5) | 0.82 |
| 7 | +1(30) | -1(10) | +1(50) | -1(34) | +1(7.5) | 1.25 |
| 8 | +1(30) | +1(30) | +1(50) | +1(40) | +1(7.5) | 2.18 |

The various factors were coded according to Eq. (1).

Letters: $x_{1}$= CSL (mL/L), $x_{2}$= molasses (g/L),$x_{3}$ = dissolved oxygen, $x_{4}$= temperature (°C) and $x_{5}$= pH value.

| **S3 Table. The variance analysis of the first-order model regression to biomass production.** | | | | | | | |
| --- | --- | --- | --- | --- | --- | --- | --- |
| Source | DF | Adj SS | Adj MS | F-Value | Effect | Coef | P-Value |
| Model | 5 | 8.37027 | 1.67405 | 3169.8 |  |  | 0.00032 |
| Linear | 5 | 8.37027 | 1.67405 | 3169.8 |  |  | 0.00032 |
| $x_{1}$ | 1 | 4.64363 | 4.64363 | 8792.67 | 1.52375 | 0.76188 | 0.00011 |
| $x_{2}$ | 1 | 2.70863 | 2.70863 | 5128.76 | 1.16375 | 0.58188 | 0.00021 |
| $x_{3}$ | 1 | 0.00015 | 0.00015 | 0.29 | 0.00875 | 0.00438 | 0.644 |
| $x_{4}$ | 1 | 0.96258 | 0.96258 | 1822.63 | 0.69375 | 0.34688 | 0.00055 |
| $x_{5}$ | 1 | 0.05528 | 0.05528 | 104.67 | -0.16625 | -0.08312 | 0.00942 |
| Error | 2 | 0.00106 | 0.00053 |  |  |  |  |
| Total | 7 | 8.37132 |  |  |  |  |  |

Letters: $x_{1}$= CSL (mL/L), $x_{2}$= molasses (g/L),$x_{3}$ = Dissolved oxygen, $x_{4}$= temperature (°C) and $x_{5}$= pH value.

| **S4 Table. The experimental design and results from the path of steepest ascent method.** | | | | | |
| --- | --- | --- | --- | --- | --- |
| Step | Variance of Factors | | | | Response |
|  | Corn steep liquor (ml/L) | Molasses (g/L) | Temperature (°C) | pH | Biomass (g/L) |
| Original point | 20 | 20 | 37 | 7 | 1.48 |
| Step 1 | 25 | 23.82 | 37.7 | 6.97 | 1.8 |
| Step 2 | 30 | 27.64 | 38.4 | 6.95 | 2.27 |
| Step 3 | 35 | 31.46 | 39.1 | 6.92 | 2.36 |
| Step 4 | 40 | 35.27 | 39.7 | 6.89 | 2.44 |
| Step 5 | 45 | 39.09 | 40.4 | 6.86 | 0.8 |

The interval between each step was determined according to the ratio of coefficients in S2 Eq. (2). The fermentation was performed in 24 hours.

**S5 Table. The cost of different medium components for *R. palustris* PS3 fermentation.**

| Composed | Purchased sources/brands | Cost of per Kg (US$) | Concentration in medium (g/L) | Cost for per liter in medium (US$) | |
| --- | --- | --- | --- | --- | --- |
|  |  |  |  | PNSB medium | Newly developed medium |
| Ammonium chloride (NH_4_Cl) | J.T. Baker Chemical Co. | 66.72 | 1 | 0.06672 | 0.06672 |
| Dipotassium phosphate (K_2_HPO_4_) | J.T. Baker Chemical Co. | 60.66 | 1 | 0.0606564 | 0.0606564 |
| Sodium chloride (NaCl) | Bio BASIC Inc. | 35.38 | 0.5 | 0.01769145 | 0.01769145 |
| Magnesium sulfate (MgSO_4_•7H_2_0) | Bio BASIC Inc. | 30.33 | 0.2 | 0.00606564 | 0.00606564 |
| Ferrous sulfate (FeSO_4_) | J.T. Baker Chemical Co. | 51.56 | 0.01 | 0.000515579 | 0.000515579 |
| Calcium chloride (CaCl_2_) | Bio BASIC Inc. | 153.80 | 0.02 | 0.003075953 | 0.003075953 |
| Manganese chloride (MnCl_2_) | Sigma-Aldrich Inc. | 454.92 | 0.002 | 0.000909846 | 0.000909846 |
| Sodium molybdate (Na_2_MoO_4_) | Sigma-Aldrich Inc. | 585.33 | 0.001 | 0.000585334 | 0.000585334 |
| Yeast extract | Bio BASIC Inc. | 70.77 | 0.5 | 0.0353829 | - |
| Malate | Alfa Aesar Co. | 67.40 | 5 | 0.33698 | - |
| Sodium acetate | KATAYAMA CHEMICAL INDUSTRIES Co., Ltd. | 22.24 | 2 | - | - |
| Peptone | Amresco | 269.58 | 5 | - | - |
| Molasses | TAIWAN SUGAR Co. Ltd. | 0.67 | 32.35 | - | 0.021802606 |
| Corn steep liquor | TAIROUN PRODUCTS Co., Ltd. | 0.61 | 39.41 | - | 0.023904687 |

**S6 Table. Quantification of total organic carbon and total nitrogen.**

|  | Newly developed *R. palustis* PS3 fermentation broth |
| --- | --- |
| Total organic carbon (g/Kg) | 30.05±0.17 |
| Total nitrogen (g/Kg) | 16.98 ±0.17 |
| C/N | 1.77 |

Bacterial fermentation was performed according the optimal culture conditions in this study.

**References**

1. Lenth, R.V. 2009. Response-surface methods in R, using rsm. J. Stat. Softw. 32(7):1-17. doi: 10.18637/jss.v032.i07

2. R Core Team. (2019). R Foundation for Statistical Computing, Vienna, Austria.

3. Nelson, D.W. and L.E. Sommers. 1982. In "Methods of soil analysis", ed. A. L. Page, pp. 539-579

4. Bremner, J.M. and C.S. Mulvaney. 1982. In "Methods of Soil Analysis Part 2 Chemical and Microbiological Properties", ed. A. L. Page, pp. 621-622. New York, USA.: Academic Press.

.
